# HOX genes in stem cells: Maintaining cellular identity and regulation of differentiation

**DOI:** 10.3389/fcell.2022.1002909

**Published:** 2022-09-13

**Authors:** Jennifer Steens, Diana Klein

**Affiliations:** Institute for Cell Biology (Cancer Research), Medical Faculty, University of Duisburg-Essen, Essen, Germany

**Keywords:** adult stem cell, mesenchymal stem cell, MSC, HSC, HOX code, differentiation, signaling pathways

## Abstract

Stem cells display a unique cell type within the body that has the capacity to self-renew and differentiate into specialized cell types. Compared to pluripotent stem cells, adult stem cells (ASC) such as mesenchymal stem cells (MSCs) and hematopoietic stem cells (HSCs) exhibit restricted differentiation capabilities that are limited to cell types typically found in the tissue of origin, which implicates that there must be a certain code or priming determined by the tissue of origin. HOX genes, a subset of homeobox genes encoding transcription factors that are generally repressed in undifferentiated pluripotent stem cells, emerged here as master regulators of cell identity and cell fate during embryogenesis, and in maintaining this positional identity throughout life as well as specifying various regional properties of respective tissues. Concurrently, intricate molecular circuits regulated by diverse stem cell-typical signaling pathways, balance stem cell maintenance, proliferation and differentiation. However, it still needs to be unraveled how stem cell-related signaling pathways establish and regulate ASC-specific HOX expression pattern with different temporal-spatial topography, known as the HOX code. This comprehensive review therefore summarizes the current knowledge of specific ASC-related HOX expression patterns and how these were integrated into stem cell-related signaling pathways. Understanding the mechanism of HOX gene regulation in stem cells may provide new ways to manipulate stem cell fate and function leading to improved and new approaches in the field of regenerative medicine.

## Introduction

Stem cells are undifferentiated cells that represent unique cell types within the body. Based on their self-renewal capacity (multiply themselves) and their differentiation potential (develop into specialized cells), stem cells are clearly essential for tissue growth and maintenance. The many different types of stem cells are formed at different times in life, and differ in the places in the body where they persist (Heins et al., 2004; Snippert and Clevers, 2011; Alvarez et al., 2012). Embryonic stem cells (ESCs) exist only at the earliest stages of development (4–7 days after fertilization) and can be extracted from the inner cell mass of the blastocyst (Evans and Kaufman, 1981; Thomson et al., 1998). ESCs are known as pluripotent stem cells, which are able to self-renew and to give rise to all cell types of the three embryonic germ layers (ectoderm, mesoderm, endoderm). Under appropriate laboratory conditions ESCs can be grown in the undifferentiated state and potentially proliferate indefinitely. As postnatal derivates of ESCs, various types of tissue-specific so-called adult stem cells (ASCs) appear during fetal development and remain as “primitive cells” in a specialized environment called niche in our bodies throughout life. ASCs are found in almost every tissue, e.g., in umbilical cord, placenta, bone marrow, larger blood vessels, lung, skin, and fat tissue (Hsu and Fuchs, 2012; Klein, 2016; Redondo et al., 2017; Cable et al., 2020; Klein, 2021). These non-reproductive “somatic” stem cells can be divided into hematopoietic stem cells (HSCs; blood stem cells), mesenchymal stem cells (MSCs) and epithelial stem cells (EpSCs) as well as neural stem cells (NSCs) (Dulak et al., 2015; Cable et al., 2020). ASCs have a high proliferative potential and -depending on their tissue of origin- the capacity to differentiate into various cell types. Compared to ESCs, the differentiation potential of ASCs is restricted, meaning multipotency (Kaebisch et al., 2015; Zakrzewski et al., 2019).

As tissue-resident stem cells, ASCs generally were important orchestrators of normal tissue homeostasis with the potential to suppress inflammation by direct or indirect cellular communications resulting in immune cell education and thus disease-specific microenvironment regulation (Bernardo and Fibbe, 2013; Planat-Benard et al., 2021). In healthy conditions, ASCs are initially considered quiescent until “activation” by organ demands, e.g. following a pathological trigger, which induces proliferation and/or migration out of their niche, and finally fosters tissue regeneration in a paracrine fashion or by differentiation to replace damaged cells (Rumman et al., 2015; Klein, 2016; Clevers and Watt, 2018; Klein, 2021; Urban and Cheung, 2021). Thus, ASCs are essential for the constant renewal of all tissues maintaining organ structure and function. Due to the enormous regenerative potential furthermore, the transplantation of isolated and *in vitro* expanded ASCs and especially MSCs has established itself as a possible strategy for the therapy of a large number of diseases, including graft-versus-host disease, lung injury, and bone diseases and defects (Cheung et al., 2020; Fu et al., 2021; Klein, 2021). The safety and feasibility of such stem cell based-based therapeutic approaches has already been confirmed in numerous clinical studies and is still the subject of current studies. However, while the exact mechanism of MSC action remains elusive, numerous preclinical studies are already showing ways to increase the therapeutic efficacy, particularly of MSCs, through specific modulations to produce exogenous stem cells with superior repair capabilities (Hu and Li, 2018; Gardin et al., 2020; Ocansey et al., 2020). Besides an inherent tropism towards inflammatory sites, MSCs also exhibit a natural tumor-trophic migration ability, suggesting that exogenous MSCs could be exploited as pathotropic delivery vehicles when loaded with bioactive anticancer drugs (Ding et al., 2021; Sentek and Klein, 2021; Takayama et al., 2021). Within these scenarios, a continuous presence of the cells does not seem to be necessary as the therapeutic potential of exogenously applied ASCs is mainly based on a “hit/kiss and run” mechanism. Recent studies also suggest that release of paracrine factors from MSCs is accomplished via so-called extracellular vesicles (EVs) such as exosomes and microvesicles (Witwer et al., 2019; Massa et al., 2020). As carriers of the whole information panel characterizing the use of ASCs for regenerative purposes, these ASC- and particularly MSC-derived EVs thus hold advantages as non-self-replicating “entities” for therapeutic applications. However, the quality of ASC-derived EVs from different sources and across batches was shown to be difficult and moreover inconsistent, which severely restricts respective quality control and management (Kou et al., 2022). In a recently published and very elegant work, the development of ‘NANOBIOME’, namely NANOmetric BIO-banked MSC-derived Exosome, is specifically proposed here for biobanking of EVs secreted by MSCs for their easy and available storage and distribution, since the use of ASC-derived EVs in particular circumvents specific biobanking problems, i.e. technical problems and regulatory concerns that have so far limited the use, for example, of MSC banking for rapid regenerative applications (Codispoti et al., 2018). Thus, regarding prospects of basic research and clinical applications, protocol standardization, including precise evaluation of samples in terms of harvesting rate, characterization as well as pre-clinical parameter assessment are worthy of attention and exploration (Codispoti et al., 2018). Within these challenges, the limited secretion of EVs from ASCs maybe a bottleneck for efficient exosome production and application, which in turn would require efficient pre-treatment strategies of ASCs for boosting the biogenesis and secretion of ASC-derived EVs (Wang et al., 2020).

**FIGURE 1:**
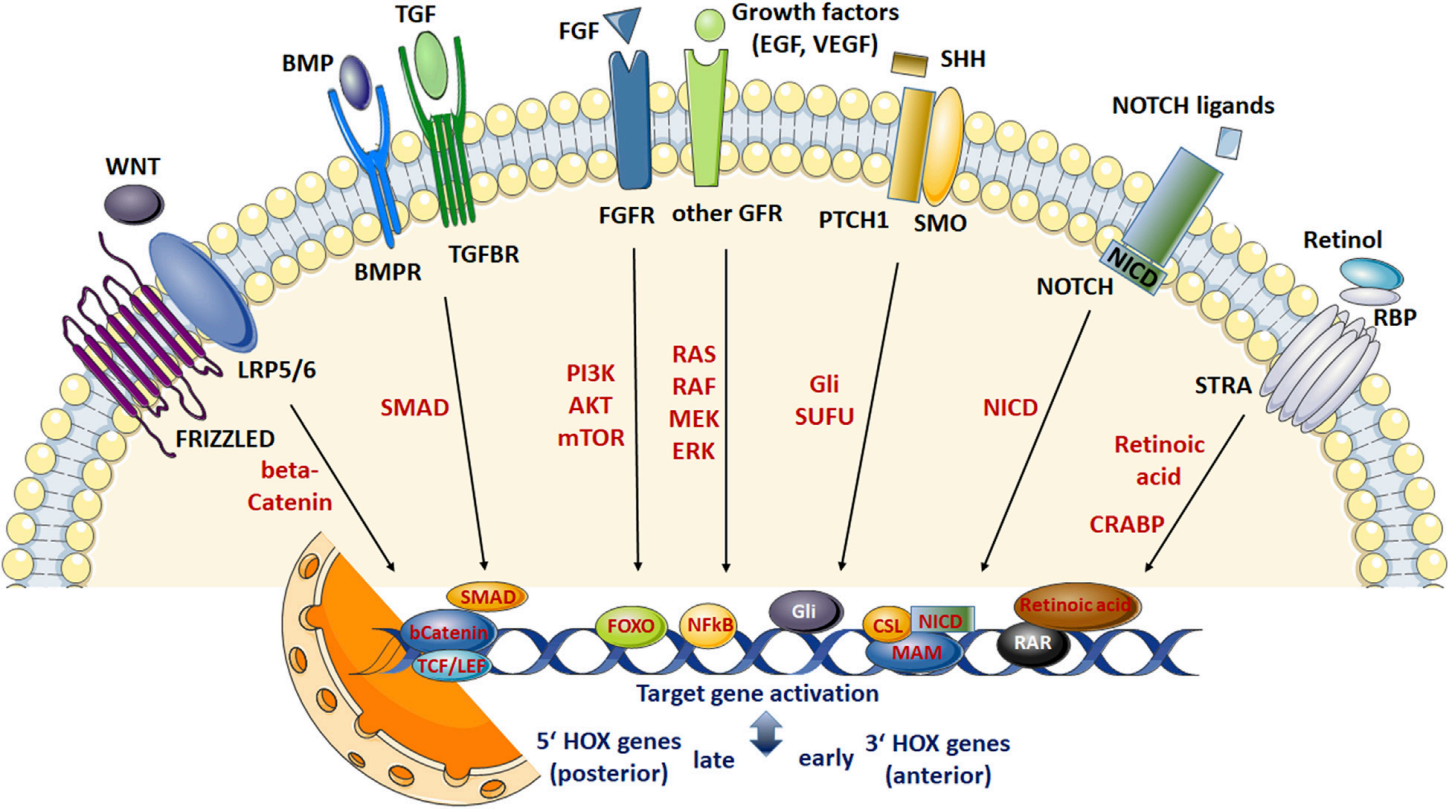
Scheme summarizing the most relevant signaling networks regulating stem cell fate. Development of adult stem cells (ASCs) as well as stem cell maintenance, proliferation, differentiation and survival require complex interactions between diverse molecular signaling pathways and downstream transduction molecules. These pathways include WNT signaling, signaling by multi-functional growth factors that belong to the transforming growth factor (TGF) beta superfamily, fibroblast growth factor (FGF) signaling, sonic hedgehog (SHH), NOTCH, and retinoic acid (RA) signaling pathways. WNT ligand binding to the receptor complex consisting of FRIZZLED and low-density lipoprotein receptor-related proteins 5/6 (LPR 5/6) results in intracellular β-catenin stabilization enabling nuclear translocation. Nuclear β-catenin then elicits gene expression changes (including HOX genes) through the T-cell factor (TCF)/lymphoid enhancer factor (LEF) family of transcription factors. Signaling pathways initiated by TGFβ ligands are transduced through cell surface receptor complexes resulting in (type I; BMPR, TGFR) receptor phosphorylation and serine-threonine phosphorylations of (effector) SMAD transcription factorsmeaning activation. Following nuclear translocation (in complexes formed with SMAD4) target gene transcriptions (including HOX genes) are activated. SMADs further can interactwith β-catenin and LEF/TCF transcriptional regulators enablingWNTsignaling in a TGFβ-dependentmanner. FGF ligands, and FGF receptors (FGFR) -like other growth factor receptors including epidermal and vascular endothelial growth factor receptors- lead to autophosphorylation of the (intracellular) protein tyrosine kinase domains and activation of various effectors such as RAS/RAF/mitogen-activated protein kinase (MAPK) and phosphatidylinositol-4,5-bisphosphate 3-kinase (PI3K)/AKT/mTOR and RAS/RAF/MAPKs finally mediating NF-kB (nuclear factor “kappa-light-chain-enhancer” of activated B-cells) or FOXO (Forkhead Box O)-dependent gene expressions (including HOX genes). Other signal transducers (Phospholipase C Gamma, PLC-γ) and activators of transcription (STAT) pathways can also be activated, which intersect and synergize with other signaling pathways, e.g.,WNT, RA and TGFβ signaling (not shown). SHHsignals through a receptor complex that includes the G-protein-coupled receptor smoothened (SMO) and the (twelve-pass)membrane protein patched 1 (PTCH1). In response to SHH ligand binding, SUFU (suppressor of fused) binding, and thus cytoplasmic sequestrations of GLI (glioma-associated oncogene family members) transcription factors become inhibited, leading to GLI stabilization and nuclear translocation resulting in SHH signal transduction, namely transcriptional activation of SHH target genes (includingHOX genes). NOTCH receptor activation results in NOTCH cleavage (through a cascade of proteolytic cleavages by ADAM metalloprotease and γ-secretase) releasing the intracellular domain of the receptor (NICD). NICD translocates to the nucleus, displaces corepressor complex, and recruits coactivators finally forming a ternary complex with the DNA binding protein CSL and the transcriptional coactivator Mastermind (MAM) to activate transcription of Notch target genes (including HOX genes). NICD can also activate the NF-κB transcription factor and thus cooperatewith growth factor signaling. All-trans RA and other active retinoids generated fromvitamin A (retinol)mediate their action by binding toRAreceptors (RAR), nuclear receptors acting as transcription factors, which are bound to DNA as a heterodimer with the retinoid X receptor (RXR) in regions called retinoic acid response elements (RAREs). The multi-transmembrane protein STRA6 was shown to mediate mediates vitamin A uptake from plasma retinol binding protein 4 (RBP4). DNA interaction of RA following nuclear transport by CRABP (cellular retinoic acid binding protein) induces transcription of genes encoding transcription factors and signaling proteins that further modify gene expression particularly early HOX genes, e.g., HOXA1 with sequential activation of the clustered HOX genes in an anterior-posterior order that resembles their positions in the chromosomal cluster. RA can also activate FOXOtranscription factors and thus cooperate with growth factor, particularly FGF signaling. Generally, it is assumed that anteriorHOXgenes locatedmore at the 3′ end of a chromosome are preferentially activated by RA pathways, while activation of posterior 5′ HOX genes are preferred by BMP and WNT signaling.

Several growth factors as well as small molecules generally orchestrate the cells signaling important for stem cell maintenance and cell-type specific functions, and signaling alterations can affect the cellular features of stem cells, particularly induce differentiation resulting in the conversion of stem cells into appropriate progenitor cells, which in turn give rise to associated cell types (Figure 1). An improved understanding of these signaling pathways and key signaling molecules for the promotion of homogenous stem cell populations and/or targeted differentiation of stem cells is not only crucial to improve stem cell function as internal repair systems of the body; the potentially manipulation of core gene networks could even improve culture conditions of respective stem cells that might allow refined generation of desired stem cell cultures and tissues with refined functional features for regenerative approaches. Signals that influence stem cell specialization processes, including the determination of cellular identity, are intimately linked to gene expression networks (Wells and Choi, 2019; Zakrzewski et al., 2019). Here, the HOX genes and HOX-regulated signaling pathways have evolved to become an integral regulator of stem cell identity and cell fate. This review therefore summarizes the latest insights how HOX gene expressions are regulated in stem cells and how HOX genes orchestrate stem cell maintenance, differentiation, and thus function.

### Signaling networks regulating stem cell fate

The formation of tissues and organs from naïve stem and progenitor cells is controlled by combinatorial signaling of certain pathways in distinct temporal windows to progressively direct embryonic cells through a series of fate decisions into specific tissue lineages. Thus, stem cell functions in adults from quiescence through “activation” resulting in self-renewal, mobilization or differentiation require tightly control by these signaling networks and complex cross-talks between the different signaling pathways (Blank et al., 2008; Denner et al., 2010; Tanabe, 2015). Fibroblast growth factor (FGF) and leukemia inhibitory factor mediated signaling turned out to be of critical importance in regulating key transcriptional regulators of pluripotency, including sex determining region Y box 2 (SOX2), octamer-binding transcription factor (OCT) 3/4, krueppel-like factor 4 (KLF4), NANOG, and c-MYC (Coutu and Galipeau, 2011; Theunissen and Jaenisch, 2014; Tanabe, 2015; Mossahebi-Mohammadi et al., 2020). Signaling pathways that further regulate cell fate decisions particularly stem cell maintenance, self-renewal and differentiation, include: (i) Wingless related integration site (WNT) molecules supposed to act as niches factors maintaining a self-renewing state (Nusse, 2008; Van Camp et al., 2014), (ii) bone morphogenetic proteins (BMPs) that belong to the transformation growth factor beta (TGFβ) superfamily critically controlling differentiation (including epithelial and mesenchymal transitions) and cell death (Wagner, 2007; Sakaki-Yumoto et al., 2013), (iii) sonic hedgehog (SHH) signaling that is an important player for ASC maintenance (Petrova and Joyner, 2014), (iv) NOTCH signaling known to be responsible for maintaining balance between the different cell fates (Chiba, 2006; Wang et al., 2009), as well as (v) small molecules such as retinoic acid (RA) that orchestrate FGF signaling to drive differentiation, and thus decisively impact on balancing of stem cell quiescence and activation (Stavridis et al., 2010; Gudas and Wagner, 2011) (Figure 1). These signaling pathways in turn modulate the expression of homeotic selector (HOX) transcription factor genes, known as master regulators of cellular fate, in a spatio-temporal manner, finally balancing stem cell maintenance, proliferation, differentiation, and survival. However, it remains be unraveled how these signaling pathways and decisive molecular pathway candidates here mediate full colinear HOX activation and enable deterministic patterning of diverse cell-type specific HOX profiles, which could potentially be used to recapitulate HOX expression profiles e.g., for the generation of target cells or for an on-site manipulation of HOX signaling alterations in diseased states.

### Homeotic selector transcription factors as master regulators of (tissue-) specific stem cells identities.

#### Establishing homeotic selector gene expression

**FIGURE 2:**
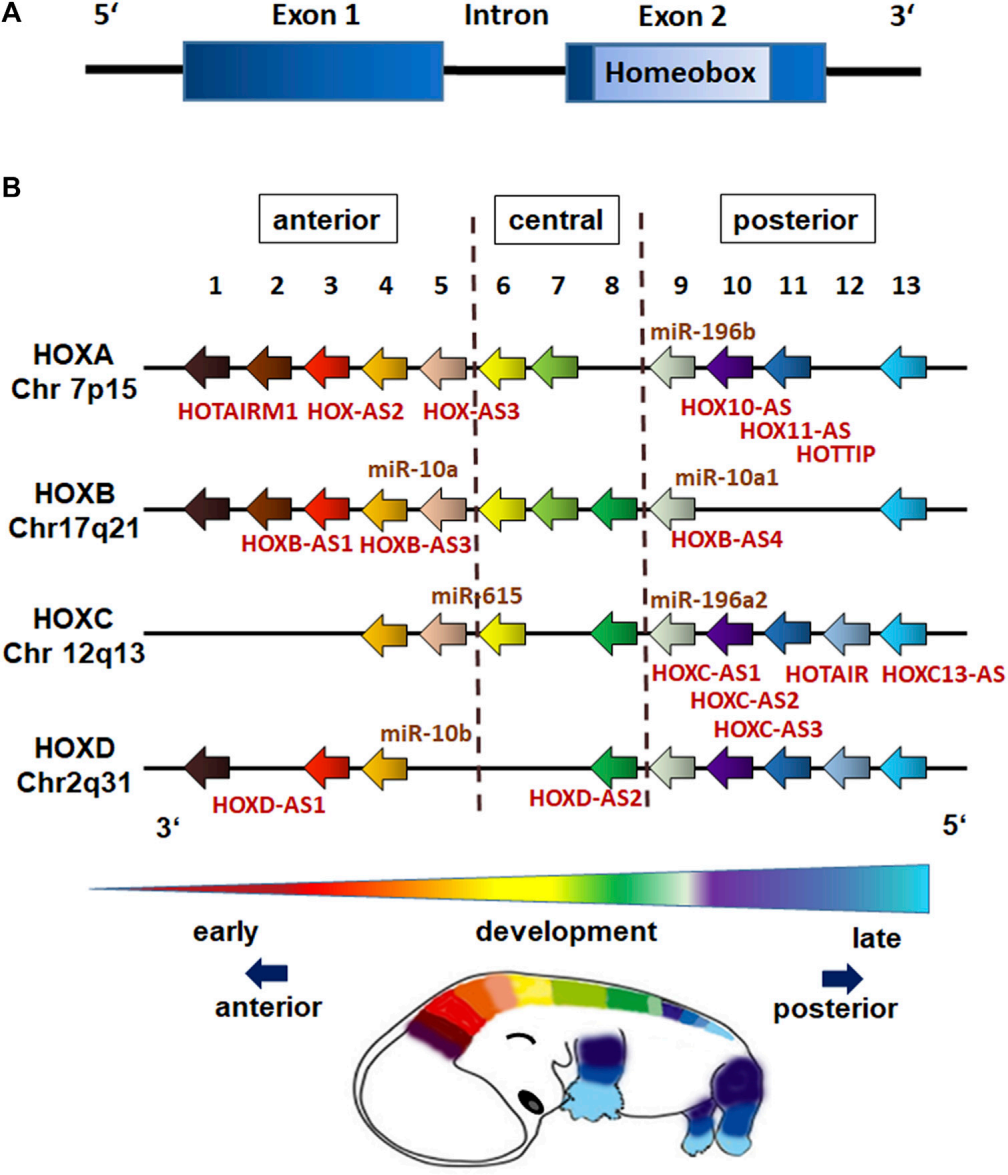
HOX gene structure and genome organization (schematic representation). **(A)** HOX genes are comprised of one intron separating two exons with the second exon having a 120-nucleotide sequence encoding for the 60 amino acid DNA-binding domain known as the homeobox (homeodomain). **(B)** The 39 human HOX genes are clustered into the four HOX families HOXA, HOXB, HOXC, and HOXD with each family consisting of nine to eleven paralogous genes (assigned by numbers based on sequence similarity and cluster position), which are responsible for the anterior-posterior specification of body segments. The position of non-coding RNAs that are interspersed within the coding HOX genes are marked (miR,microRNAs; AS, antisense RNAs). HOX gene expressions exhibit spatial and temporal collinearity: nested domains of HOX genes are generated with anteriorly HOX expressions operating earlier in development and posteriorly HOX expressions occurring later.

HOX genes comprise a family of highly conserved, regulatory genes that encode transcription factors, which control the activity of other, functionally contiguous genes in the context of morphogenesis. Beside a number of other gene families that include position-relevant information for body construction (e.g., PAX and T-BOX genes), the main task of HOX genes is structuring the embryo along the body longitudinal axis (Figure 1). HOX genes are named after the homeobox, a evolutionarily conserved 180 nucleotide pairs long sequence encoding for the homeodomain (Figure 2A), a DNA binding motif allowing respective HOX proteins to negatively or positively regulate target gene expressions (Krumlauf, 1994; Ruddle et al., 1994). In vertebrates, HOX genes were found to be arranged in four clusters (A–D) with the known 39 human HOX genes being located on chromosome 7q, 17q, 12q and 2q (Ruddle et al., 1994). The order of the HOX genes on a chromosome corresponds to the time expressing the body sections controlled by them and follows HOX gene expression along the anterior-posterior axis (Figure 2B). This means that HOX genes controlling the development of terminal body segments are also at the end of this gene group on the chromosome (Harding et al., 1985; Kmita and Duboule, 2003; Seo et al., 2004). During embryonic development HOX genes become activated following the temporal sequence in 3′-5′direction (temporal collinearity) and correlating with the gene position (spatial collinearity) (Gaunt, 2015; Durston, 2019a).

HOX gene expression is generally coordinated by transcriptional regulation of HOX genes from earlier segmentation genes and cross-regulatory interactions among HOX genes (Morata and Kerridge, 1982; Irish et al., 1989). 3′ anterior HOX genes are expressed first and can activate expression of 5′posterior HOX genes, a regulation called posterior induction (PI) (Durston, 2019b). Posterior HOX genes in turn are able to suppress anterior ones at functional level, a phenomenon called posterior prevalence (PP) (Akam, 1987). The newer concept of posterior dominance (PD) nowadays describes the regulation of HOX expressions at mRNA level (Durston, 2019b). HOX proteins bind to small AT rich base regions, often with the same TAAT core sequence (Noyes et al., 2008). HOX-DNA-binding specificities are further achieved by cooperatively binding cofactors such as TALE homeodomain cofactor proteins PBX and MEIS, and by HOX collaborators, namely proteins that bind in parallel to HOX-targeted cis-regulatory modules (CRMs) (Mann et al., 2009; Slattery et al., 2011; Sanchez-Higueras et al., 2019). An integration of growth factors and signaling molecules such as RA, FGFs, BMPs and WNTs, which are expressed in gradients along the embryonic axis further determine the restricted domains of HOX expression (Diez del Corral and Storey, 2004; Darras et al., 2018; Nolte et al., 2019; Afzal and Krumlauf, 2022). Small non-coding RNAs (ncRNAs) such as microRNAs and the long ncRNAs (lncRNA) can further impact on HOX gene expressions (Figure 2B). LncRNAs for example were shown to determine an epigenetic profile of HOX loci by association with Polycomb Group (PcG) and Trithorax Group (ThrxG) proteins (Rinn et al., 2007). The lncRNA HOTAIR, which is located within the HOXC locus, takes part in silencing of HOXD genes by interacting with polycomb repressive complex 2 (PRC2) and thus complex formation finally leading to histone H3 lysine 27 trimethylation (H3K27me3) of the HOXD locus to repress genes (Rinn et al., 2007). In contrast, the lncRNA HOTTIP that is located at the 5′end of the HOXA locus was shown to coordinate the activation of various 5′ HOXA genes (Wang et al., 2011). Mechanistically, HOTTIP directly binds to the adapter protein WDR5, which is a component of the mixed lineage leukemia complex methylating lysine 4 of histone H3 across the HOXA locus leading to histone H3 lysine 4 trimethylation (H3K4), and thus gene activation (Wang et al., 2011). Generally, histone H3 methylations leading to an active H3K4 and a repressive H3K27 mark (with H3K27me3 being dominant over the H3K4me3) decisively regulate HOX expression patterns. During gastrulation, trimethylation of H3K27 is induced by PRC2, which recruits PRC1 leading to inhibition of chromatin remodeling activity and chromatin condensation to maintain silencing of HOX genes (Rea et al., 2000; Soshnikova and Duboule, 2008). Demethylation of H3K27 in turn can induce the expression of the hitherto repressed (lineage-specific) HOX genes (Bernstein et al., 2006; Soshnikova and Duboule, 2008). These chromosomal domains marked either by an active H3K4 or silent H3K27 turned out to be crucial to establish an epigenetic memory (including the HOX genes) of the cellular identity in stem cells and moreover following differentiation (Ringrose and Paro, 2007; Kim et al., 2010; Ezziane, 2012). The simultaneous expression of a certain combination of HOX genes (termed the “HOX-code”) further on was presented to be tissue-specific (Kessel and Gruss, 1991), as HOX genes impose positional identity to developing tissues (Mallo et al., 2010). In adults, this segmental or particularly HOX memory persists in downstream tissue-resident stem cells (Kamkar et al., 2016; Smith et al., 2019). Accordingly, stem cells exhibit heterogeneous but characteristic HOX expression profiles that are highly specific for their anatomical origin, and are maintained during differentiation (Ackema and Charite, 2008; Liedtke et al., 2010; Klein et al., 2013; Kamkar et al., 2016; Smith et al., 2019). Together with the fact that nested domains of HOX expression arise in part through the ability of HOX clusters to integrate and respond to certain signaling gradients, it is important to understand these interactions, and how decisive regulatory mechanisms through which signaling pathways coordinately control the precise HOX expression patterns that are required for specifying diverse stem cell features including morphology (Afzal and Krumlauf, 2022).

### The homeotic selector code

#### Homeotic selector gene expression in pluripotent stem cells

Pluripotent stem cells are characterized by the capabilities to self-renew and to differentiate into the three primary germ cell layers during early embryogenesis, and therefore to generate all cells of the adult body. Within humans, OCT4, SOX2 and the homeobox transcription factor NANOG are considered to be the core nuclear transcription factors that regulate pluripotency, particularly in ESCs (Chambers et al., 2003; Boyer et al., 2005; Young, 2011). Other ESC characteristic markers, which are used to define, characterize and to isolate ESCs are the cell surface antigens stage-specific embryonic antigen-1 (SSEA1/CD15), SSEA3, SSEA4, as well as TRA-1-60 (Podocalyxin/TRA-1-81), and signal pathway-related intracellular markers like the signal transducer SMAD2/3, the TGFβ/Activin/Nodal signal pathway, transcription activators like the WNT/β-catenin signaling pathway, as well as the enzymatic marker alkaline phosphatase (Zhao et al., 2012). As an “*in vitro* alternative”, nonembryonic, so-called induced pluripotent stem cells (iPSCs) reprogrammed from somatic cells (Takahashi and Yamanaka, 2006; Takahashi et al., 2007) have been established, which have the same pluripotent characteristics than ESCs but lack ethical concerns raised about the use of embryos for ESC isolation (Choi et al., 2015; De Los Angeles et al., 2015; Volarevic et al., 2018).

As HOX genes are not expressed before gastrulation, HOX genes were not found to be transcribed in ESCs (Deschamps and Duboule, 2017). Pluripotent stem cells in general were shown to harbor an active epigenetic repression of HOX genes in this undifferentiated, naïve state (Ezziane, 2012; Luo et al., 2019; Smith et al., 2019). Herein, HOX gene promoters often exhibit both H3K4me3 and H3K27me3 marks, and thereby HOX genes remain in a dormant state waiting for activation (Soshnikova and Duboule, 2008). Upon differentiation, histone demethylases such as UTX (lysine-specific demethylase 6A, known as ubiquitously transcribed tetratricopeptide repeat, X chromosome) and JMJD3 (Jumonji domain-containing protein-3) are recruited to remove repressive marks on H3K27 from HOX lineage-specific genes, while transcriptional repression of HOX genes being specific for other lineages are maintained by PcGs (Agger et al., 2007; Riising et al., 2014). Similarly, so-called unrestricted somatic stem cells (USSCs) from human cord blood that exhibit a broad ecto-, meso-, and endodermal differentiation potential were characterized by the absence of HOX expressions (Liedtke et al., 2010; Santourlidis et al., 2011). Although USSCs lack presence of the major stem cell factors OCT4, SOX2, and NANOG, an epigenetic state in between that of an ESC and of a terminally differentiated cell was reported enabling USSCs to exhibit differentiation and reprogramming cues typical for pluripotent stem cells (Santourlidis et al., 2011). In the course of the differentiation process tendencies for HOX profiles become then more prominent and refined. However, it needs still to be unraveled which HOX code exactly commits pluripotent stem cells to a certain lineage. The recent discoveries of HOX profiles specific to ASCs, thus orchestrating multipotent stem cells through differentiation and tissue-specific maturation processes are now summarized.

### Homeotic selector gene expression in adult stem cells

**FIGURE 3:**
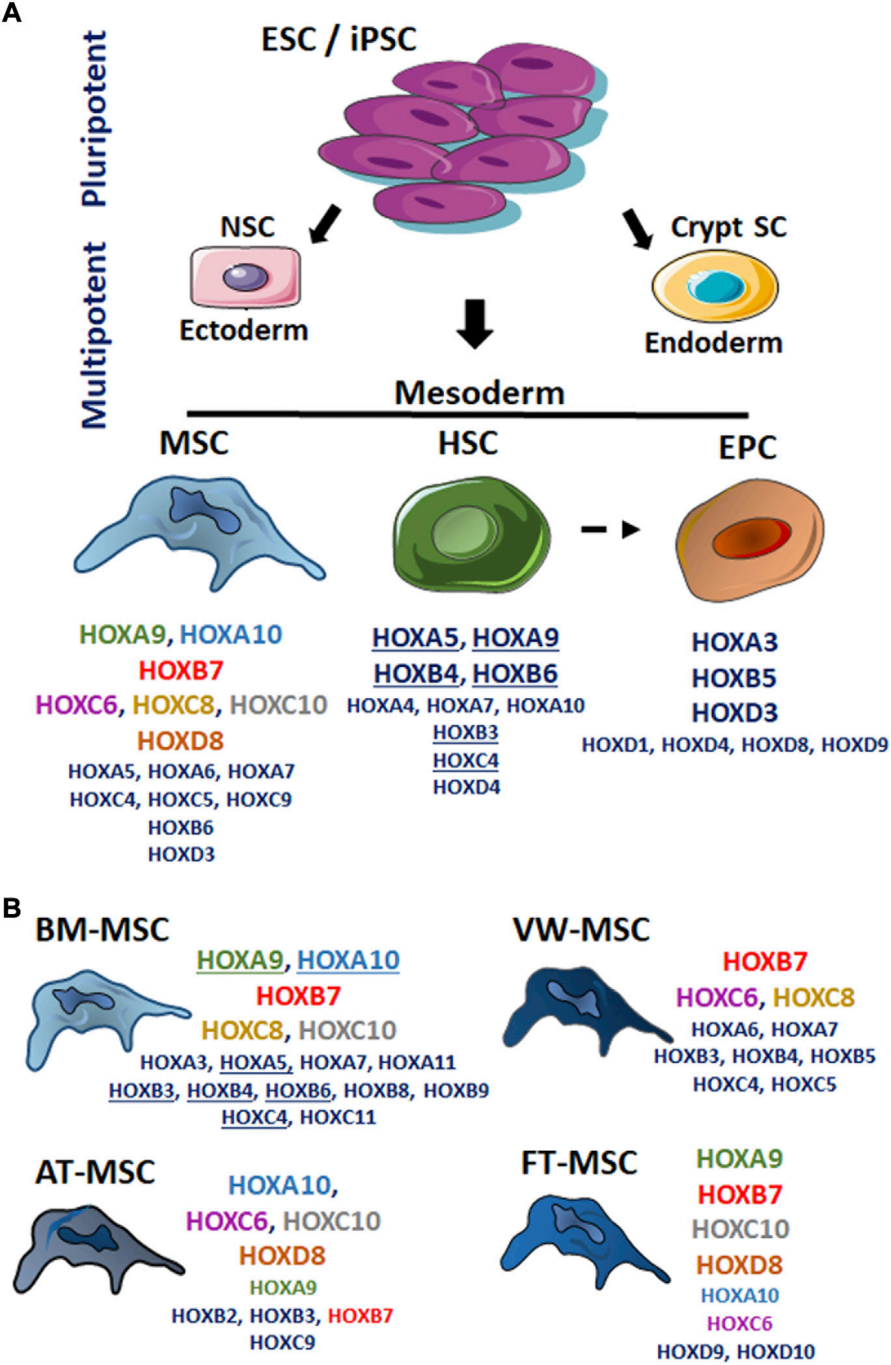
The HOX code of mesodermal stem cells. Stem cells derived from different tissues present patterns of HOX gene expression (“the HOX code”) that mirrors their developmental origin. According to the fact that HOX genes are not expressed before gastrulation, HOX genes were not found to be transcribed in non-differentiated, pluripotent stem cells (SC) due to active epigenetic repression of HOX genes. In cells, particularly stem cells lying at equivalent anteroposterior positions but in distinct embryonic germ layers, HOX proteins have distinct regulatory activities. **(A)** The reported HOX expression pattern for the mesodermal-derived adult stem cell types were listened: mesenchymal stem cell (MSC), hematopoietic stem cell (HSC) and endothelial progenitor cell (EPC). (For details: see main text.) **(B)** HOX expression pattern for bone marrow (BM)-, vascular wall (VW-), adipose tissue (AT)-, and fetal tissue (FT; summarizing umbilical cord, placenta and amniotic fluid MSCs)-MSCs were separately shown. Capital letters name more frequently identified HOX genes potentially representing the cell type-specific HOX code, whereas smaller letters designate individual additionally identified HOX genes above it. Same colors highlight same HOX genes that are common in different MSC types; underlined HOX genes emphasize similar HOX genes between BM-MSCs and HSCs. ESC, embryonic stem cell; iPSC, induced pluripotent stem cell; NSC neural stem cell.

The positional identity that emerged from HOX gene expression patterns during embryonic development was found to be maintained in many adult tissues, particularly in ASCs, suggesting that the topographic specificity of these HOX codes as an intrinsic property is maintained during differentiation, and thus provides a mechanism for imposing cell identity and fate restriction (Yamamoto et al., 2003; Smith et al., 2019). Indeed, ASCs were found to exhibit characteristic HOX expression signatures that are heterogeneous but highly specific for their anatomical origin (Figure 3). The persistent expression of specific HOX genes in many adult tissues indicates that, in addition to the cellular identity, the function of these cells and respective organ tissues containing them is also determined by HOX genes meaning that HOX genes were co-opted for location-specific functions (Wellik, 2009; Krumlauf and Ahn, 2013; Quinonez and Innis, 2014). Due to the large number of findings in the ASCs, the following focus concerning stem cell-type specific HOX gene expression pattern and how this is related to stem cell function is rather on mesodermal stem cells: MSCs, HSCs and endothelial progenitor cells (EPCs).

### Homeotic selector gene expression in mesenchymal stem cells

Human MSCs can be isolated and expanded from almost every organ, preferred however from bone marrow, peripheral blood and various neonatal birth-associated tissues, blood vessels, and adipose tissue. Although these MSCs were phenotypically highly similar, MSC cultures are generally quite heterogenous. According to the minimal MSC criteria (as defined by the International Society of Cell Therapy) *in vitro* expanded MSC must adhere on plastic, express the MSC markers CD73, CD90 and CD105 while lacking expression of hematopoietic and endothelial lineage markers, and exhibit the capability of *in vitro* differentiation into adipocyte, osteoblast, and chondrocyte lineages (Dominici et al., 2006; Viswanathan et al., 2019).

Compared gene expression profiling of MSCs derived from bone marrow (BM), adipose tissue and cord blood identified 25 well-characterized genes including the two HOX genes HOXA5 and HOXB6 in all MSC preparations, irrespective of origin and culture conditions (Wagner et al., 2006). However, none of these genes alone was specific for MSCs and the definition of a unique marker, particularly a HOX gene or a HOX gene constellation, for MSCs remained elusive (Wagner et al., 2006; Ho et al., 2008). Investigations using murine MSCs (identified as colony forming unit-fibroblasts) from different organs firstly revealed that MSCs of different origins -although phenotypically highly similar- can be distinguished by their specific topographic HOX code depending on the tissue of origin (Ackema and Charite, 2008). Consistent with the assumption that the HOX code is an intrinsic property of ASCs, it was hypothesized that the MSC-type specific HOX code is part of a “blueprint” required for the tissue-specific (regenerative) action of MSCs (Ackema and Charite, 2008). Accordingly, it was suggested that MSC-specific HOX expression patterns as “biological fingerprint” could be used to distinguish human MSC populations of functionally distinct tissues (Liedtke et al., 2010). HOXA9, HOXB7, HOXC10 and HOXD8 (and to a lesser extend HOXA10 and HOXC6) were defined as potential molecular markers, which are highly expressed in fetal MSC derived from cord blood (CB-MSCs; compared to USSC) (Jansen et al., 2010; Liedtke et al., 2010). The HOXA cluster here turned out to be highly methylated, whereas the HOXB-D clusters are less methylated indicating that HOX genes from these clusters (HOXB6,7, HOXC4-10, HOXD3,4) are involved in CB-MSC maintenance and function (Liedtke et al., 2010). BM-MSCs showed a highly similar HOX code compared to CB-MSCs identifying HOXD9 and HOXD10 (being absent in BM-MSC) as decisive HOX genes enabling distinction (Liedtke et al., 2010). HOXC10 (being present in Decidua-derived MSCs) was further defined as a potential marker to distinguish amnion- and decidua-derived MSCs (Hwang et al., 2009). And HOXC10 (together with T-box 15 transcription factor) seemed to be a fundamental developmental transcription factor in adipose tissue-derived MSCs (AT-MSCs) (AD-MSCs) (Onate et al., 2013; Miklosz et al., 2022). Furthermore, increased expressions of HOXA10, HOXC6, HOXD8, and to a lesser extend of HOXA9, HOXB2, HOXB3, HOXC9, HOXC10 were reported in these cells (Kouidhi et al., 2015). BM-MSCs, as one of the commonly used sources for MSCs, usually show increased expressions of HOXA9, HOXA10, HOXB4, HOXB7, HOXC8, HOXC10, and HOXD8 (Coenen et al., 2015). By comparing different human BM-MSCs, namely MSCs isolated from bone marrow isolated from iliac crest, sternum, and vertebrae, it was shown that BM-MSCs at all express HOX genes in high numbers (35/39), with the different BM-MSC populations expressing specific sets of HOX genes in increased levels (Picchi et al., 2013). HOXC10 and HOXC11 were found to be upregulated in iliac crest-MSCs, HOXB8 and HOXB9 in vertebrae-MSCs, and HOXA3, HOXA5, HOXB6, HOXB7, HOXB9, HOXC4 as well as HOXC8 in sternum-MSCs (Picchi et al., 2013). Besides, so-called vascular wall-derived MSCs (VW-MSCs), which are located within the vascular stem cell niche, in the so-called “vasculogenic zone” of the blood vessel wall, were characterized by increased expression levels of HOXB7, HOXC6 and HOXC8; but also HOXA6, HOXA7, HOXB3, HOXB4 and HOXB5 as well as HOXC4 and HOXC5 expression can be detected (Klein et al., 2013). Even lung resident MSCs (LR-MSCs) were shown to express the VW-MSC specific HOX code of HOXB7, HOXC6 and HOXC8 (together with HOXB5), as these cells were found to be predominately located with the vascular adventitial stem cell niche (Klein, 2021; Steens et al., 2021). Accordingly, LR-MSCs turned out to be phenotypically and functionally indistinguishable from VW-MSC, a fact that further highlights the HOX code as master regulator of cellular identity.

Taken together, a distinct expression of HOXA9 and HOXA10, HOXB7, HOXC6, HOXC8, and HOXC10, as well as HOXD8 can frequently be observed in MSCs derived from different human tissues, and thus in certain combinations- potentially represent the MSC-type specific HOX code (Figure 3). It can also be stated that mainly central HOX genes, or HOX genes that are in the immediate vicinity of the central cluster region are expressed within tissue-specific MSCs. But besides their central role in defining segment identity, ensuring that the right structures are formed in the right body places, the activity HOX genes as well as their expression levels control segment-specific structures and cell types. Herein, other less frequently reported HOX genes, e.g. HOXA5, HOXA6 and HOXA7, HOXB6, HOXC4, HOXC5, and HOXC9, as well as HOXD3 might be important contributors to these actions.

The most common and reliable way to identify MSCs (in addition to surface marker analysis), is to verify their trilineage differentiation potential into adipocytes, osteoblasts, and chondrocytes *in vivo* and *in vitro*. HOX genes were of functional importance in regulating these differentiation processes [reviewed in (Seifert et al., 2015)]. As revealed from early mouse studies for example, the HOX candidate HOXC8 was shown to critically regulate the progression of cells along the chondrogenic differentiation pathway (Yueh et al., 1998). Particularly in MSCs, HOXC8 expressions were shown to be upregulated during chondrogenic differentiation and a fostered HOXC8 expression caused an enhanced expression of chondrogenic markers, promoted the chondrogenic differentiation and the formation of cartilage clumps (Yang et al., 2020a), while osteogenic differentiation was suppressed (Yang et al., 2020b). A forced expression of HOXC8 was even associated with adipogenesis inhibition (Mori et al., 2012). As another example, HOXA11 and HOXD11 functioned in regulating (the early steps) of chondrocyte differentiation (Gross et al., 2012). Loss of function studies further revealed that HOX11 impairments in MSCs of the bone marrow (and periosteum) at adult stages caused defects in differentiation, leading to an overall deficit in the cartilage production and thus defects in endochondral ossification (Rux et al., 2016). And in certain MSC types, namely in regional skeletal MSCs, which arose from the earliest stages of skeletal development, HOX11 expression turned out to be decisive for their self-renewal and for functioning as progenitors for osteoblasts, chondrocytes and adipocytes throughout lifetime (Pineault et al., 2019). Particularly HOXA cluster genes seem to play a central role here, because HOXA cluster negative cord blood MSCs failed to differentiate properly into the chondro-osteogenic lineages with the HOX candidates HOXA2 and HOXA10 being of special meaning (Liedtke et al., 2017). Herein, HOXA10 in BM-MSCs was already shown enabling RUNX2 activation, a central regulator of osteogenesis (Hassan et al., 2007). Mechanistically HOXA10 mediated chromatin hyperacetylation and H3K4 methylation of the bone-related RUNX2 P1 promoter by interacting through a HOX core motif, that caused activation of early osteogenic genes through the chromatin remodeling (Hassan et al., 2007). HOXC10 was further identified as a direct target of the lncRNA lncHOXC-AS3, which stabilized HOXC10 expression in BM-MSCs thereby regulating osteogenesis (Li et al., 2019).

Thus, it is clear that HOX genes exert MSC-specific functions particularly concerning the trilineage potential along mesodermal lineages. However, the observed differences of HOX genes in regulating similar processes maybe based in the difference of HOX codes orchestrating different but phenotypical highly similar MSC types, which in turn dependent of the tissue of origin. Interestingly, HOXB7 turned out to be an “universal MSC” HOX gene as it was reported to be expressed in all the different types of MSCs (Liedtke et al., 2010; Klein et al., 2013; Foppiani et al., 2019). An induced expression of HOXB7 was identified as a master player driving proliferation and differentiation of human BM- and AT-MSCs (Foppiani et al., 2019; Casari et al., 2021), and accounts for the high proliferation and sprouting potential of VW-MSCs (Klein et al., 2013; Klein, 2016). However, rather than accounting for stem cell type-specific functions, HOX genes specify various regional properties of a tissue along the rostral-caudal axis by regulating a wide range of cellular activities such as proliferation and differentiation, cellular adhesion, migration and invasion, as well as cell death (Parrish et al., 2009). The persisting HOX expression profiles of the different tissue-specific MSCs strongly suggest that those specific HOX profiles are of particular importance for the functioning of tissues throughout adult life with resident HOX-expressing MSCs as leading regulators of embedding stroma renewal and regeneration (Kulebyakina and Makarevich, 2020). Following a rough classification along the cranial-caudal direction, HOX1-4 paralogues were shown to act predominately in cranial tissues, followed by HOX5-6 in subsequent upper sternal tissues, and HOX7-8 in lower sternal and abdominal tissues, whereas the paralogues HOX9-13 represent HOX genes building up HOX codes in caudal body parts and extremities (Kulebyakina and Makarevich, 2020). An example for the association of HOX gene expression within the organization of tissue-specific structures and features comes from VW-MSCs (Klein et al., 2013). Under normal tissue homeostasis (“quiesence”), when residing in the adventitial vasculogenic stem cell niche, these cells express the HOX pattern HOXB7, HOXC6 and HOXC8 at high level that were shown to suppress the expression of transgelin (TAGLN), a TGFβ-inducible gene-inducible gene found to be essentiell for early smooth muscle cell differentiation. Upon commitment, VW-MSCs progress through tissue-specific differentiation events, where a silencing of the VW-specific HOX genes altered the TAGLN promotor methylation sites finally causing increased TAGLN expression, which induced then VW-MSC differentiation into tissue-typical smooth muscle cells (Klein et al., 2013).

Thus, modulation of cell-type specific HOX expression levels as well as respective patterns might offer potential druggable targets for innovative on-site tissue regeneration approaches or for the improved generation of stem cells *in vitro*, particularly of MSCs with superior repair capabilities. Within that scenario, enforcing trans-differentiation of pluripotent stem cells or even other somatic cell types towards MSCs by ectopic expression of HOX genes as MSC-specific transcription factors turned out to be a straight forward approach to generate MSCs *in vitro* in huge numbers desirable especially for regenerative purposes (Steens and Klein, 2018; Abdal Dayem et al., 2019). The *in vitro* generation of vascular wall-typical MSCs from (murine) iPSCs, based on a VW-wall MSC-specific HOX code was already reported (Klein et al., 2013; Steens et al., 2017; Steens et al., 2020a). A lentiviral vector expressing a small set of identified (human) VW-MSCs-specific HOX genes, namely HOXB7, HOXC6 and HOXC8 was here used to directly program murine iPSCs into MSCs, which then displayed classical MSC characteristics, both *in vitro* and *in vivo.* As HOX selector genes are highly conserved throughout evolution, it is assumed that forced expression of this HOX code also leads to MSC differentiation from human iPSCs, although the final proof for human iPSCs remains to be shown (Steens et al., 2017; Steens et al., 2020a). It could be shown accordingly, that the same triple combination of HOX genes as VW-MSC-specific gene code was sufficient to directly convert human skin fibroblasts towards MSCs, and thus directing cell fate conversion bypassing pluripotency (Steens et al., 2020b). The introduced HOX-code in primary human skin fibroblasts could further be linked to an increased colony formation and trilineage differentiation potential as (mesenchymal) stem cell characteristics (Steens et al., 2020b). However, it remains to be clarified whether the triple HOX combination approach of HOXB7, HOXC6 and HOXC8 actually retains the VW-MSC-specific master regulatory function to generate VW-MSCs, or whether the induced expression of individual (HOXB7, HOXC6 or HOX candidates already could serve as MSC-specific key transcription factor. Corresponding investigations could also shed light on which MSC characteristic(s) depend(s) on which introduced HOX gene.

### Homeotic selector expressions in hematopoietic stem cells and endothelial progenitor cells

HSCs can be classically found in the bone marrow and the peripheral blood being prerequisite for sustained hematopoietic reconstitution and the formation of blood cells. Whereas HSCs were known to be derived from mesodermal precursor cells called hemangioblasts during embryogenesis, HSC in adults (as identified phenotypically by tyrosine-protein kinase c-KIT/CD117 and stem cell antigen-1 (SCA1) expressions, while being lineage negative and by exhibiting functional hemangioblast activity) serve as a rich source for circulating EPCs in addition to the generation of blood cells (Cogle and Scott, 2004). During adult life, EPCs have been defined by their cell surface expression of the hematopoietic marker proteins CD133 and CD34 and the endothelial marker vascular endothelial growth factor receptor-2 (VEGFR2), and their capacity to incorporate into sites of neovascularization prior *in situ* differentiation into endothelial cells; thus, being decisive for new vessel formation (Asahara and Kawamoto, 2004; Ishige-Wada et al., 2016; Yoder, 2018). HSCs as well as their derived hematopoietic progenitors were known to be characterized by HOX genes in a pattern characteristic of the lineage and stage of differentiation of the cell (He et al., 2011), although total HOX expression levels of HSCs were suggested to be rather low (Bijl et al., 2006; Ackema and Charite, 2008).

The vigorous ability of HSC to produce huge numbers of lineage differentiated cells includes erythrocytes, (mega-) karyocytes, innate and acquired immune cells (Haas et al., 2018). And hematopoiesis is critically impacted by HOX gene expressions: anterior HOX genes (comprising the numbers 1–6 of the 3′ region) are maximally expressed in the more primitive HSCs, whereas posterior HOX genes (comprising the numbers 7–13 of the 5′ region) become prominent in the committed progenitors (Sauvageau et al., 1994; He et al., 2011). Quantification of HOX gene expression levels in the different hematopoietic cell populations isolated from human peripheral blood revealed that all these cells generally express HOXA and HOXC cluster genes at significantly higher levels compared to expression levels HOXB and HOXD cluster genes (10–100-fold lower) (Morgan and Whiting, 2008). High HOXA5 expression levels for example were suggested to account for hematopoietic lineage determination being able to promote differentiation along myelopoietic lineages (Fuller et al., 1999; Argiropoulos and Humphries, 2007). HOXB3, HOXB4, and HOXA9 were shown to play a crucial role for the presence of HSCs, as a combined deficiency in these HOX genes fostered severe effects on hematopoietic organs (Magnusson et al., 2007). Furthermore, the HOX candidates HOXA9, HOXB4, and HOXB6 were highly expressed in HSCs (Seifert et al., 2015; Bhatlekar et al., 2018). Particularly high HOXB4 expression levels seemed to be important for most potent HSCs, namely long-term reconstitution HSCs (Wang et al., 2005; Fan et al., 2012; Forrester and Jackson, 2012). A forced HOXB4 expression was shown to generate HSCs *in vitro*, and to account for the proliferative response of long-term repopulating HSCs as well as for HSC maintenance (Brun et al., 2004; Pilat et al., 2005). And, the other way around, a reduction of HOXB4 led to a reduction of HSC proliferation (and differentiation) and, to a reduction of neighboring HOX genes in an 3′to 5′order namely of HOXB2, HOXB3, HOXB5 (Brun et al., 2004). Similarly, HOXA4 and the HOX paralog group 4 at all were shown to be decisive for a functional HSC phenotype (Iacovino et al., 2009). Besides, HOXA5, HOXA9, and HOXA10 were identified as three of seven transcription factors (together with ERG, LCOR, RUNX1 and SPI1) to convert “pre-differentiated” tissue, namely haemogenic endothelium cells into HSCs (Sugimura et al., 2017; Fidanza and Forrester, 2021). Studies from the *in vitro* generation of HSCs using human pluripotent stem cells further revealed that HOXA cluster genes were significantly downregulated in (CD34-positive) HSCs that were incapable of long-term engraftment and repopulation, strongly indicating that HOXA genes critically regulate definitive hematopoiesis (Ng et al., 2016). Particularly, increased HOXA5 and HOXA7 expression levels were associated with the repopulation activity (Dou et al., 2016). In accordance with the 3′ to 5′ HOX direction, the HOX candidate HOXA9 then together with the ETS-family transcription factor ERG were further shown to re-specify lineage-restricted (CD34^−^ and CD45-positive) HSC precursors derived from human iPSCs into primitive (CD34-positive and CD38-negative) HSCs (Doulatov et al., 2013). Thus, stimulation of HOXA gene expression potentially improves HSC maintenance generating self-renewing HSCs from pluripotent stem cells (Collins and Thompson, 2018).

Decreases in HOX gene expressions were then observed upon HSC differentiation in a manner that seems to follow their anterior-posterior position with anterior HOX genes being downregulated earlier than posterior HOX genes (Guo et al., 2003; He et al., 2011). Generally, HOXB and single HOXC cluster genes were associated to hematopoietic cells with erythroid features, and HOXA cluster genes with myeloid features (Lawrence et al., 1996; He et al., 2011). Within that scenario, HOXA5 and HOXA9 were shown to be involved in the proliferation and differentiation of HSCs to common myeloid progenitors, with HOXA9 also regulating the differentiation of HSCs into common lymphoid progenitors (Bhatlekar et al., 2018). During the differentiation (of pre-B cells) into B cells, HOXB3 was found to be a relevant factor, while HOXC3 and HOXC4 crucially impacted on erythroid lineage differentiation (Bhatlekar et al., 2018). HOXA5 and HOXC8 further seemed to regulate erythroid differentiation of megakaryocyte-erythrocyte progenitors, with HOXA7 being expressed during megakaryocyte differentiation (Bhatlekar et al., 2018).

Conclusively, HOX gene expression does not only specify HSC identity, HOX genes significantly impact in HSC function as well as in stages of hematopoietic differentiation; and dysregulation of these HOX genes were associated with a number of leukocyte malignancies (as discussed elsewhere: (McGonigle et al., 2008; Alharbi et al., 2013)). However, a precise activation of indicated HOX genes following stimulation of different pathways orchestrating HSC characteristics were not reported up to now (Demirci et al., 2020). Thus, the sequential activation pattern of HOX genes following signaling induced HSC differentiation remains to be unraveled.

Although a number of transcriptomic profiles and EPCs characterization are available, the role of HOX genes in EPCs known to be involved in neovascularization processes remains nearly completely elusive (Medina et al., 2010; Wu et al., 2018; Abdelgawad et al., 2021). This may be due, at least in part, to the fact that many different cell subtypes are consistently grouped together under the term “EPC”, which in turn would argue in favor of setting minimum criteria for defining EPCs, e.g., detailed immunophenotyping and/or potency assays, similar to defining minimal criteria for characterizing MSCs (Medina et al., 2017). However, several HOX transcription factors were already associated with target gene expression known to promote the differentiation of mature endothelial cells, which might indicate that also EPCs exhibit a specific HOX code. HOXB5 might be an EPC HOX code candidate, as HOXB5 was involved in the *in vitro* differentiation of embryonic precursor cells towards the endothelial lineage (Wu et al., 2003). Endothelial cell differentiation of ESCs further showed that HOXA3 and HOXD3 are immediately expressed when differentiation is induced, whereas HOXA5 and HOXD10 are expressed in more mature and adult endothelial cells (Bahrami et al., 2011). Accordingly, high expressions of HOXD3 together with HOXD1, HOXD4, HOXD8 and HOXD9 were reported for less mature blood-derived outgrowth endothelial cells (Toshner et al., 2014). HOXD3 was further linked to endothelial activation (from a resting to an angiogenic state) (Boudreau et al., 1997), and HOXB3 seemed to be required for subsequent capillary morphogenesis (Myers et al., 2000). And HOXA13 was found to be essential for placental vascular patterning and labyrinth endothelial specification (Shaut et al., 2008). HOX genes involvement in endothelial differentiation was further revealed when the HOX expression profiles of BM-MSCs were investigated following endothelial differentiation (Chung et al., 2009). The expression patterns of the four HOX genes HOXA7, HOXB3, HOXA3, and HOXB13, significantly changed during endothelial cell differentiation with expression levels of HOXA7 and HOXB3 becoming increased, whereas those of HOXA3 and HOXB13 became decreased. According to the central role of HOXA9 for the cellular identity of HSCs, HOXA9 was shown to mediate maturation of endothelial cells and being a master switch to regulate the expression of typical endothelial-committed genes such as endothelial nitric oxide synthase, VEGFR2, and VE-cadherin (Rossig et al., 2005). HOXA9-deficiency was further reported to result in reduced EPCs numbers and thereby impaired the postnatal neovascularization capacity (Rossig et al., 2005). Likewise, reduced HOXA9 expression levels were estimated in CD34-positive cells of hypertensive patients, an effect that correlated with reduced EPC numbers (Pirro et al., 2007). EPCs isolated from umbilical cord blood showed that HOXD9 seemed to be required for EPC maintenance (Iordache et al., 2015). Thus, several HOX genes, especially HOX genes from HOXA and HOXD cluster, take part in EPC maintenance, endothelial cell differentiation and function, however an EPC-specific HOX code could not be defined up to now. It seems that within endothelial differentiation processes a complex interaction of various HOX genes are important. EPC or endothelial cell-specific HOX codes, and how respective HOX genes then contribute to EPC-endothelial cell functions as well as to the proper functioning of the adult vasculature need to be unraveled.

### Signaling networks regulating stem cell properties by modulating homeotic selector gene regulation

The diverse molecular signaling pathways and dependent transduction molecules known to orchestrate various stem cell characteristics regulate and control HOX gene expression. In addition to the involvement of HOX genes in the positional identity of stem cells along the stem cell hierarchy, HOX genes decisively function in all stem cell characteristics: self-renewal and maintenance, proliferation and survival, migration and invasion and lineage specification (differentiation). However, less is known how the main signaling pathways operating in stem cells regulate HOX expressions, and how single HOX transcription factors in turn impact on stem cell-related signaling pathways, and thus account for certain stem cell functions. Now we summarize the recent knowledge how the important stem cell-related signaling pathways such as WNT, BMP, FGF, SHH, NOTCH and RA, integrate HOX genes for maintaining cellular identity and regulation of differentiation.

Generally, during human embryogenesis, more 3′ located (anterior) HOX genes (HOX1-8) become activated and regulate paraxial mesoderm development with the formation of embryonic primordial segments called somites by modulating cell ingression into the primitive streak (Iimura and Pourquie, 2006). Subsequently activated more 5′ located (posterior) HOX genes (HOX10-13) limit mesoderm ingression by repressing WNT signaling, and mediating body axis termination. (Young et al., 2009; Denans et al., 2015). Concerning the signaling it is known that the paraxial regions of the mesoderm become specified following the action of BMPs, members of the FGF family and WNT molecules. Cross-regulation of these pathways, e.g., by the WNT effector molecule NOGGIN that inhibits BMP signaling, is not only important for the paraxial mesoderm specification but also for maintaining this identity (Aulehla and Pourquie, 2010; Budjan et al., 2022). As a model of human somitogenesis, paraxial mesoderm organoids were developed by differentiating human pluripotent stem cells towards paraxial mesoderm using a combination of WNT signaling activation together with BMP inhibition and following FGF2 treatment (Budjan et al., 2022). Thereby, the sequential activation pattern of the HOX genes was elegantly recapitulated. HOXA1 expression was early detected following WNT activation and BMP inhibition (with 24 h), followed by other cervical (HOX1-HOX5 paralogues) and thoracic (HOX6-HOX9 paralogues) HOX genes within the next 48–72 h, to HOXD9, a lumbosacral HOX gene in the somite-stage organoids of day 4–5, which was 24–48 h after the successive FGF2 treatment (Budjan et al., 2022). Concerning the mechanism it was revealed that WNT molecules activate HOX gene expression in a temporally collinear way as WNT-dependent enhancers, namely interaction regions with HOXA1 called “HOXA developmental early side (ADES)” that are located within the posterior region. WNT signals (particularly WNT3) caused thereby removal of H3K27 repressive marks on the 3′ region of the HOXA cluster that in turn promotes transcription of HOXA genes (Neijts et al., 2016). Subsequently to initial WNT-induced activation of 3′ HOX genes, coactivated caudal type homeobox genes (Cdx genes) were shown to act as crucial effectors for expression induction of central and 5′ HOX genes in a collinear manner (Neijts et al., 2017).

RA (a vitamin A metabolite)-dependent signaling is another central signaling pathway being essential for early embryonic development and the promotion of stem cell lineage specifications in pluripotent stem cells (Soprano et al., 2007; Gudas and Wagner, 2011). Studying the kinetics of transcriptional and epigenomic patterning responses of RA in ESCs revealed that RA plays even an essential role in HOX gene regulation by binding to specific retinoic acid receptors (NR1Bs) at RA responsive DNA elements (RAREs) located within the HOX clusters finally erasing H3K27me3 from repressed HOX genes during ESC differentiation (Kashyap et al., 2011). RA stimulation generally induces activation and recruitment of demethylases to HOX genes, which in turn become demethylated thereby promoting HOX expression and thus differentiation (Shahhoseini et al., 2013). Genes at the 3′ end of the complexes (e.g., the paralogs HOXA1, HOXB1, and HOXD1) display herein a higher responsiveness to RA while genes at the 5’ end (the paralogs HOXA13, HOXB13, HOXC13, and HOXD13) are more responsive to FGF signaling (Nolte et al., 2019). FGF signaling was shown to interact antagonistically with RA signaling during posterior development, finally resulting in morphogen gradients within the anterior-posterior-axis with high RA concentrations at the anterior and high FGF concentrations at the posterior end, which lead to an increased expression of the HOX genes HOXB9 and HOXA7 (Kumar et al., 2021).

Thus, stem cell fate decisions into specific tissue lineages require distinct temporal windows for the known stem cell-related signaling pathways and respective cross-regulations (Rankin et al., 2018). On the one hand, FGF-related signaling was shown to orchestrate the pluripotent stage (Mossahebi-Mohammadi et al., 2020). FGF family members, namely FGF2 and FGF4 were shown to function as intrinsic regulators of pluripotent stem cell self-renewal and survival by promoting MAPK and AKT signaling pathways. Particularly MAPK signaling seems to be required, whereas PI3K/AKT signals increase as pluripotency gets restricted (Lee et al., 2019; Mossahebi-Mohammadi et al., 2020). Moreover, FGF-related signaling being essential for pluripotency maintenance, especially FGF4 expression, depends on the presence of the pluripotency transcription factors OCT4 (and SOX2). On the other hand, FGF-related signaling, particularly through FGF2, has even the potential to induce lineage differentiation of pluripotent cells (Steens and Klein, 2018; Ma et al., 2019). An FGF2 activation was found to activate the downstream kinase Src, a non-receptor tyrosine kinase that in turn activates MEK1*/*2 (Mitogen-activated protein kinase 1) resulting in differentiation and/or increased proliferation particularly of mesodermal ASCs (Ma et al., 2019). Very recently it was shown that respective processes here were linked to HOX gene regulation (Tiana et al., 2022). The pluripotency factor OCT4 turned out to be important for maintaining HOX genes silent in the stage of pluripotency. Upon lineage commitment however, OCT4 switches from a repressor to an activator being required for the proper activation of HOX genes. Especially the genes of the HOXB cluster are coordinately regulated by OCT4 binding sites located at the 3′ end of the cluster (Tiana et al., 2022).

Concerning the time of induced HOX expressions, so how extrinsic factors influence the tempo of HOX expressions, it was recently shown that the progressive activation of HOX genes in pluripotent stem cells is controlled by a dynamic increase in FGF signaling (Mouilleau et al., 2021). Herein, an increase in FGF pathway activity was successfully associated with the sequential activation of HOX genes; and cells differentiated under accelerated HOX induction (Mouilleau et al., 2021). Combination of “cell type-specific” growth factors then are able to foster lineage specifications and thus further modulation of HOX expressions. Pluripotent stem cell differentiation towards mesodermal stem cells for example can be initiated by the induction of mesodermal differentiation, achieved by TGFβ/Activin/Nodal signaling inhibition (Mahmood et al., 2010). Subsequent treatment with cell type-specific growth factors particularly FGF2 and/or platelet-derived growth factor A/B, two known potent inducers of MSC differentiation under defined cell-culture conditions foster then MSCs differentiation (Steens and Klein, 2018; Abdal Dayem et al., 2019). For HSCs, VEGF together with hematopoietic cytokine cocktails as type-specific growth factors were generally used to drive hematopoiesis following mesoderm specification of pluripotent stem cells by the use of WNT agonists and BMP4 (Sturgeon et al., 2014; Alsayegh et al., 2019). However, reports are lacking, which follow the timely expression of mesodermal stem cell-related HOX genes induced by extrinsic signals. In contrast, it has become well established that deregulated signaling pathways and thus abnormal signal transduction as selective characteristics common to many cancers, are associated with deregulation of HOX genes. Increasing numbers of reports here revealed important molecular signaling interactions, mainly due to up-regulations of certain HOX candidates that finally result in cancer (stem cell) overpopulation, limited differentiation, and cellular disorganization of respective cancer tissues. For example, in HOXB7-overexpressing tumors, which were shown to be enriched in gene signatures classically characterizing ASCs as well as pluripotent stem cells, the pluripotency factor LIN28B was identified that sustained the expansion of a subpopulation of cells with stem cell characteristics (Monterisi et al., 2018). As another example, HOXC8 was shown to function as transcriptional activator of TGFβ signaling, finally mediating increased proliferation, anchorage-independent growth and migration of lung cancer cells (Liu et al., 2018). A more systematic evaluation of all human HOX gene target pathways being involved in biological processes associated with the cancer hallmarks revealed then, that five HOX genes (HOXC4, HOXB2, HOXB3, HOXC6, and HOXA13) and their respective targets contribute to sustained proliferative signaling (Brotto et al., 2020). As cancers are commonly characterized by an abnormal signal transduction associated with increased proliferation (and loss of apoptosis), the identification of the signal(s) that cause upregulation of certain HOX genes is still missing. It is generally assumed that a tumor-specific loss of control over the spatiotemporal expression pattern and levels compared to the expression pattern of related normal tissues occurs. Induced “tumor” HOX genes can also occur due to so-called gene dominance, when respective HOX gene expressions in normal tissues are missing. In addition, epigenetic changes in regulatory regions of HOX genes can lead to a loss of normal control (Abate-Shen, 2002; Brotto et al., 2020). The deregulated expression of HOX gene candidates however turned out to be cancer-type dependent and even show variations in respective clinical responses. Thus, unraveling the driving forces of HOX gene dysregulations may foster the development of new and additional therapeutic targets to improve cancer therapy. Within these scenarios, data sharing through the so-called blockchain technology is becoming increasingly necessary, which would improve not only the insights of researchers and clinicians; it could also serve as a suitable basis for an efficient and effective evidence-based decision-making process in therapeutic approaches, as reasoned from other diseases (Dubovitskaya et al., 2020; Fusco et al., 2020). For the current state of knowledge on the roles of HOX genes in human cancer the following review articles were recommended (Bhatlekar et al., 2018; Smith et al., 2019; Paco et al., 2020; Feng et al., 2021).

## Conclusion

The positional identity provided by HOX gene expression that arose during development and being maintained in ASCs can be linked to a limited number of stem cell typical signaling pathways in the different ASCs. On the one hand, HOX gene expression pattern (“HOX codes”) become more and more established in ASCs, particularly for MSCs. The HOX genes HOXA9 and HOXA10, HOXB7, HOXC6, HOXC8, and HOXC10, as well as HOXD8 in certain combinations potentially built up the MSC-type specific HOX codes as these HOX candidates are frequently observed in human MSCs derived from tissues different sources. Among them, HOXB7 was identified as a master player driving MSC proliferations, whereas the HOXC cluster candidates were shown to be involved in the (trilineage) differentiation capabilities. HOXA9 and HOXA10 were further identified as relevant candidates contributing to the ASC phenotype of HSCs, and being regulators of hematopoietic differentiation, whereas the additional HSC HOX code candidate HOXB4 decisively impacts on HSC maintenance and expansion. At the same time there are known extrinsic factors that can be used to generate ASCs at least *in vitro*; but how these signaling factors definitely account for a given HOX identity, and what is the respective timing of these factors remains to be unraveled. Genetically engineered HOX gene expressions hold great promise to manipulate and improve stem cell functions, particularly to promote differentiation into desired lineages, which in turn would allow then manufacturing exogenous stem cells or tissues with superior repair capabilities *in vitro*, particularly for regenerative purposes. Modified, mostly induced gene expression of individual HOX genes and particularly their overexpression can be functionally linked to tumor development and progression. However, it is not yet understood how a HOX candidate, which is widely expressed in many tissues, regulates different target genes in a cell-type-specific manner, and which interactions with additional co-activators, co-repressors and sequence-specific transcription factors predominate that are involved in, and thus could presumably be used to, model cell type-specific transcription outcomes. The identification of crucial HOX upstream regulators for individual HOX genes or HOX gene “partners” as well as their respective targets in a given cell specification process remain urgently needed, which in turn would allow generating desired ASCs without the use of genetic material that generally limits their clinical uses. An improved understanding of HOX gene functions and their respective regulations in ASCs would even foster the development of innovative strategies for a potential on-site manipulation of these cells, or particularly their dysregulated HOX gene expression as found in many cancers, directly within their endogenous niche.
